# Intelligent Transducer for Temperature Measurement with Two-Wire or Three-Wire Platinum RTD

**DOI:** 10.3390/s24237689

**Published:** 2024-11-30

**Authors:** Wiesław Miczulski, Mariusz Krajewski, Sergiusz Sienkowski, Elżbieta Kawecka, Andrzej Perec

**Affiliations:** 1 Institute of Metrology, Electronics and Computer Science, University of Zielona Góra, Szafrana 2, 65-246 Zielona Góra, Poland; w.miczulski@imei.uz.zgora.pl (W.M.); m.krajewski@imei.uz.zgora.pl (M.K.); 2 Faculty of Technology, Jacob of Paradies University, Chopina 52, 66-400 Gorzów Wielkopolski, Poland; ekawecka@ajp.edu.pl (E.K.); aperec@ajp.edu.pl (A.P.)

**Keywords:** accurate transducer, temperature measurement, RTD, auto-calibration, Monte Carlo method

## Abstract

The article presents an intelligent temperature transducer (ITT), which can work with a two-wire or a three-wire platinum resistance temperature detector (RTD). The ITT design allowed for compensation of the RTD’s lead wire resistance. The ITT used the author’s auto-calibration procedure, which minimized linearity errors of the ITT and RTD processing characteristics, ITT offset and gain errors, and errors resulting from changes in the ITT operating conditions concerning the nominal conditions. The presented results of a simulation and experimental studies confirmed the high effectiveness of this procedure. The determined uncertainty of temperature measurement using the Monte Carlo method and the obtained experimental results confirmed the possibility of measuring temperatures in the range of 0–200 °C with an expanded uncertainty of 0.02 °C at a 99% confidence level.

## 1. Introduction

Temperature is one of the most popular non-electrical quantities in terms of the number of measurements performed. It can be measured by non-contact and contact methods. Non-contact measurement can be realized, for example, with infrared thermometers and thermal imaging cameras [1,2,3]. Contact measurement is most often carried out with various types of electrical sensors. Among them, the resistance temperature detector (RTD) made of platinum (Pt) can be distinguished due to its relatively good metrological and operational properties. Its parameters are specified in the standard [4]. RTDs are manufactured, among others, by using microtechnology [5,6,7,8]. They are used primarily in monitoring industrial processes [6,9,10] and the environment [8,11,12]. In particular, the work in [6] concerns the measurement of liquid temperature in a pipeline, while [9] addresses temperature measurement in a vessel. In [10], the authors suggest the use of an RTD for modern industries and process control plants. In turn, in [8], biodegradable detectors are presented, which can be used for environmental measurements. RTDs can also be used in weather stations [11] and smart homes [12].

Temperature measurements using RTDs are carried out using temperature transducers (TTs), which process the sensor resistance into voltage (*R_S_*/*V*) [13,14,15,16,17,18,19] or into time (*R_S_*/*t*) [10,20,21]. Next, the voltage (*V*) or the time (*t*) are most often converted in TTs into a digital signal related to the temperature measured using an analog-to-digital converter (A/D) or a counter, respectively. TTs with *R_S_*/*V* circuits are more popular due to the higher accuracy of temperature measurements achieved. A Wheatstone bridge [13,16,17] or a resistive divider [14,15,18,22] is used as an *R_S_*/*V* circuit. Regardless of the circuit used for signal processing in TTs, the RTD accuracy specified in the IEC-60751 standard [4] must be considered, which depends on the class and measurement range of the given RTD. The TT processing error should be significantly smaller than the RTD error. The accuracy of the TT is influenced by its parameters, such as nonlinearity, gain and zero errors, and accuracy of supply voltage and resistors used in an *R_S_*/*V* circuit. Additionally, the nonlinearity of the RTD characteristic and the resistance of the RTD wire can have a significant influence on the temperature measurement result.

The RTD linearity error can be minimized by using the calibration methods presented in [23,24,25,26]. To achieve a temperature measurement accuracy of hundredths or even tenths of a degree, it is necessary to minimize the influence of wire resistance. Therefore, circuit solutions with three-wire and four-wire RTDs are often used [12,15,22,27,28,29,30,31,32]. In measurement circuits cooperating with a four-wire detector, the lead wire resistance has a negligible effect on the temperature measurement result. In circuits with a three-wire detector, compensation for the lead wire resistance is used. In the case of a two-wire RTD, measurement circuits are often used that do not minimize the influence of wire resistance, and the temperature measurement accuracy is the lowest. However, it is possible to compensate for the lead wire resistance of a two-wire detector [28,33,34]. One compensation method uses a two-wire RTD with an additional compensation loop (the sensor connection is sometimes referred to in the literature as a four-wire connection) [13,28,35]. It involves appropriately introducing an additional wire (compensating loop) into a measurement circuit with a resistance equal to the resistance of the two lead wires of the RTD.

Ensuring high accuracy of temperature measurement requires the use of appropriate hardware and software solutions that allow for minimizing the remaining processing errors. However, when analyzing the previously mentioned papers on TTs with *R_S_*/*V* circuits, it should be stated that not all processing errors are minimized in the above-mentioned works.

Solutions are also used to achieve very high accuracy of temperature measurements, assuming that some of the processing errors, such as linearity and gain errors, offset voltages, and errors resulting from the influence of ambient temperature, are very small. This assumption can be met at a constant ambient temperature by the additional use of very precise amplifiers, stable power sources for an *R_S_*/*V* circuit, and A/D converters with very high resolution in TTs [29,36,37].

One of the objectives of this paper is to fill the research gap regarding the application of the author’s auto-calibration procedure [38] to other temperature transducer designs. In the work in [13], the authors presented the first version of a temperature transducer based on a Wheatstone bridge circuit and an auto-calibration circuit consisting of five standard resistors. A certain limitation of this solution is the relatively complex structure of this measuring circuit and the difficult software implementation of the auto-calibration procedure. In this paper, a new circuit solution of an intelligent temperature transducer circuit is presented for which the auto-calibration procedure [38] is applied. This new solution was based on a resistive voltage divider circuit [14,22], which consisted of an RDT and a resistor connected in series. In the auto-calibration circuit, it was proposed to use four standard resistors. The use of an auto-calibration procedure in this transducer enabled the automatic minimization of RTD nonlinearity, an *R_S_*/*V* circuit nonlinearity, offset, gain errors, and errors resulting from changes in the supply voltage and ITT operating conditions concerning the nominal conditions. For this reason, this solution did not require the use of A/D converters with very small gain error and offset, and very accurate power sources, in the measurement circuit. The new ITT was dedicated to cooperation with a two-wire RTD with a compensation loop or a three-wire RTD. The advantages of the new intelligent temperature transducer, compared with the one presented in [13], are a simpler design, easier implementation of the auto-calibration procedure [38], and higher accuracy of the *T* measurement.

The article consists of four sections and one appendix. Section 2 presents the ITT concept, including its circuit diagram, general operating principle, and metrological properties. Section 3 shows an exemplary ITT implementation, including materials and simulation and experimental results. Section 4 contains the conclusions. Appendix A presents a comparison of the test results of the new ITT with the results obtained for a transducer from [13].

## 2. The Concept of an Intelligent Temperature Transducer

### 2.1. Circuit Diagram and General Principle of Operation of the ITT

**Figure 1:**
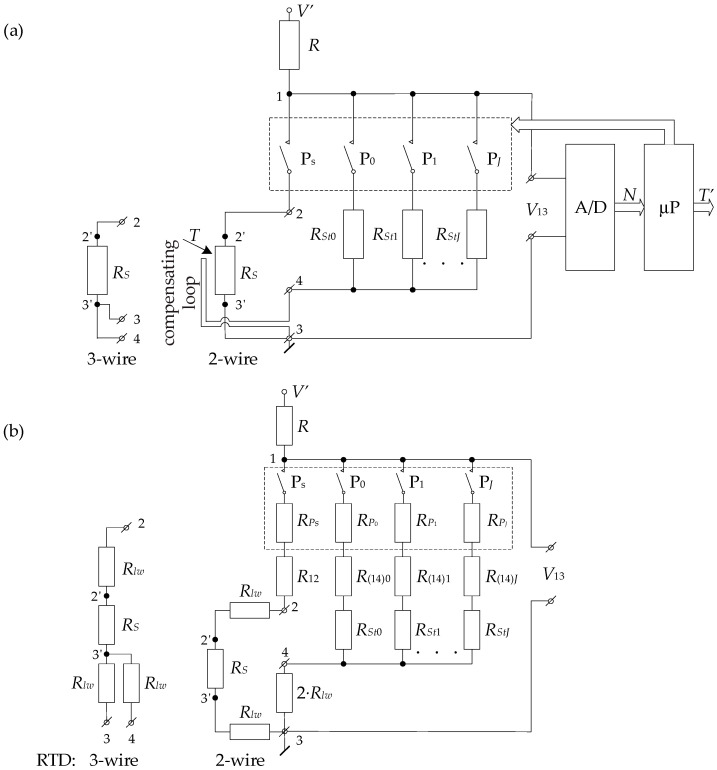
ITT circuit diagram (**a**) and equivalent circuit diagram of its *R_s_*/*V*_13_ and auto-calibration circuits (**b**).

Figure 1a shows a diagram of the ITT circuit that can work with two- and three-wire RTDs, whose wires have the same resistance. The ITT allows compensation for the detector’s lead wire resistance using a compensation loop. The RTDs are connected to the ITT at points 2, 3, and 4. The two-wire detector is connected to points 2 and 3, and the compensation loop is connected to points 3 and 4. In this case, the compensation loop is made of the same wires of the same length as the detector’s two lead wires. In turn, a three-wire RTD is connected without a separate loop to points 2, 3, and 4. Then the two lead wires of the RTD, connected to points 3 and 4, constitute a compensation loop.

The ITT with an RTD (Figure 1a) consists of four basic circuits. The first two circuits, whose equivalent diagram is shown in Figure 1b, are as follows:

- A circuit for processing the RTD resistance *R_S_* into voltage *V*_13_*S*_, consisting of a P_S_ switch, which is powered from the voltage source *V′* through a resistor *R*: The value of the *V*_13_*S*_ voltage is determined by the formula

(1) $$V_{13\_S}=V\prime \frac{R_{S}+R_{l\_S}}{R+R_{S}+R_{l\_S}},$$

where *R_l_*__*S*_ = *R*_12_ + 2*R_lw_* + *R_Ps_* is the resistance of the line connecting the *R_S_* with points 1–3 in the ITT. In this, *R*_12_ is the sum of the connection resistance of the P_S_ switch with points 1 and 2, *R_Ps_* is the resistance of the P_S_ switch in the on state, and 2*R_lw_* is the resistance of the lead wire between points 2–2′ and 3–3′. In this circuit, the resistance *R* and the supply voltage *V′* should be selected so that for the maximum resistance *R_S_* (corresponding to the maximum measured temperature), the voltage *V*_13_*S*_ is matched (close to but not greater) to the range of the A/D converter, which is located in a further part of the ITT. In addition, *R* and *V′* should be selected so that the current flowing through the *R_S_* detector does not cause a significant influence of the detector’s self-heating on the temperature measurement result. It was assumed in this study that this current should not exceed 1 mA. For this current value, the measurement range 0–200 °C, and the mean self-heating coefficient value *E_K_* = 0.15 °C/mW [39], the temperature measurement error caused by self-heating of the Pt1000 detector does not exceed 0.15 m°C.

- An auto-calibration circuit, powered by a voltage source *V′* through a resistor *R*, containing standard resistors (from *R_St_*_0_ to *R_StJ_*): These resistors are switched on accordingly by switches (P_0_…P*_J_*). The number of standard resistors and their resistance values depend on the adopted degree (*J*) of the polynomial describing the processing characteristics of the entire measurement circuit (Figure 1), as discussed further in this section. A compensation loop is included in series with the standard resistor *R_Stj_* (*j* = 0…*J*) between points 3 and 4, whose task is to compensate the sum of the connection line resistance between points 2–2′ and 3–3′ (resistance of the RTD sensor wires) [28]. The *V*_13_*Stj*_ voltage value is determined by the equation

(2) $$V_{13\_Stj}=V\prime \frac{R_{Stj}+R_{l\_Stj}}{R+R_{Stj}+R_{l\_Stj}},$$

where *R_l_*__*Stj*_ = *R*_(14)*j*_ + 2*R_lw_* + *R_Pj_* is the resistance of the line connecting the *R_Stj_* with points 1–3 in the ITT. In this, *R*_(14)*j*_ is the sum of the resistances of the connection of switch P*_j_* and *R_St_*_j_ with points 1–4, *R_Pj_* is the resistance of the P*_j_* switch in the on state, and 2*R_lw_* is the resistance between points 3 and 4. For the three-wire RTD, as a result of the auto-calibration procedure, the wire resistances (*R_lw_*) between points 3–3′ and 4–3′ (Figure 1) naturally form a compensation loop.

The other ITT circuits are as follows:

- An analog-to-digital (A/D) converter that converts *V*_13_ analog voltages to a digital representation;
- A microprocessor (μP) that properly controls the operation of the *R_S_*/*V*_13_*S*_ and auto-calibration circuits and calculates the value of the measured temperature (*T′*).

The purpose of the auto-calibration procedure is to minimize errors (linearity of RTD and *R_S_*/*V*_13_ circuits, gain error, offset voltage, and supply voltage changes) caused by changes in *T* measurement conditions from the nominal conditions. The procedure is carried out in two stages. In the first stage of the ITT auto-calibration procedure, the voltages *V*_13_*S*_ and *V*_13_*Stj*_ (for the successively switched on reference resistors *R_Stj_* and RTD (*R_S_*)) are converted by the A/D converter to obtain the digital values *N_S_* and *N_St_*_0_, *N_St_*_1_, *N_St_*_2_,…, *N_StJ_*. Then these values are stored in the µP memory. In the second stage of the auto-calibration procedure, the saved digital values together with the specified values of standard temperatures *T_Stj_* constitute the basis for calculating the current values of the polynomial coefficients from *a*_0_ to *a_J_*, describing the characteristics of the entire processing path, from the equations [38]

(3) $$\begin{array}{l} a_{0}+a_{1}T_{St0}+a_{2}T_{St0}^{2}+\ldots +a_{J}T_{St0}^{J}=N_{St0},\\ a_{0}+a_{1}T_{St1}+a_{2}T_{St1}^{2}+\ldots +a_{J}T_{St1}^{J}=N_{St1},\\ a_{0}+a_{1}T_{St2}+a_{2}T_{St2}^{2}+\ldots +a_{J}T_{St2}^{J}=N_{St2},\\ \ldots \ldots \ldots \ldots \ldots \ldots \ldots \ldots \ldots \ldots \ldots \ldots \ldots \ldots \ldots \ldots \ldots \ldots \ldots \ldots \\ \ldots \ldots \ldots \ldots \ldots \ldots \ldots \ldots \ldots \ldots \ldots \ldots \ldots \ldots \ldots \ldots \ldots \ldots \ldots \ldots \\ a_{0}+a_{1}T_{StJ}+a_{2}T_{StJ}^{2}+\ldots +a_{J}T_{StJ}^{J}=N_{StJ}. \end{array}$$

Standard temperatures *T_Stj_* are values calculated based on the RTD characteristic (*R_S_* = *f*(*T*)—detector resistance as a function of the ambient temperature in which it is located) presented in the standard [4] for the resistors *R_Stj_* used. The values of the coefficients *a*_0_, *a*_1_,…, *a_J_* also depend on external factors affecting the parameters of the components used in the ITT and on the failure to meet the condition [13]:

(4) $$R_{l\_Stj}=R_{l\_S}.$$

Then, based on the calculated values of the coefficients from *a*_0_ to *a_J_* and the measured value of *N_S_*, a non-linear equation [38],

(5) $$a_{0}+a_{1}T\prime +a_{2}T\prime^{2}+\ldots +a_{J}T\prime^{J}=N_{S},$$

is solved in microprocessor (μP), the result of which is the value of the measured temperature *T′*.

The degree of the polynomial (5), describing the processing characteristics of the transducer’s measurement track, indicates the number of standard resistors needed in the measurement circuit. The number of these resistors (and thus the degree of the polynomial) affects the accuracy of the temperature determination *T′*, among others, under the nominal conditions of the transducer. Typically, an increase in the number of resistors increases the accuracy of the temperature determination under nominal conditions but leads to a more complex measurement circuit and more difficult mathematical operations to solve Equation (5). In general, the values of the standard resistors are determined based on initially assumed standard temperatures, distributed evenly over the entire measurement range. For these temperatures, the resistances are calculated using the formula *R_S_* = *f*(*T*) [4], and then, based on these resistances, resistors are selected from a range of values available in the market.

Generally, the result of the determined temperature can be written

(6) $$T=T\prime \pm \Delta_{T},$$

where Δ*_T_* is the error of the measured temperature *T′*, which depends on the following:

- The adopted number of standard resistors and their values, accuracy (tolerances), and temperature coefficients (TCRs);
- Condition (4) is satisfied;
- The resolution and nonlinearity of the A/D converter and distortion in the form of noise [29] occurring in the ITT.

The tolerance of the resistor *R* and the zero, gain, and power ITT errors do not significantly influence the temperature measurement results by using of the auto-calibration procedure. The following part of the article discusses temperature measurement errors associated exclusively with the metrological properties of the ITT. The RTD accuracy class is not considered in the temperature measurement accuracy evaluation.

### 2.2. ITT Properties

#### 2.2.1. Effectiveness of Minimizing the Influence of Linearity, Zero, Gain, and Power Supply Errors on Temperature Measurement Results 

In order to show the effectiveness of the auto-calibration procedure and comparing the metrological properties of the ITT with the transducer presented in [13], the following assumptions were made:

- The temperature measurement range was 0–200 °C.
- RTD (Pt1000) resistance was determined by the formula [4]

(7) $$R_{S}=R_{S0}\left(1+aT+bT^{2}\right),$$

where *R_S_*_0_ = 1000 Ω, *a* = 3.9083·10^–3^ °C^−1^, and *b* = −5.774·10^–7^ °C^−2^.

- In the simulation process, the temperature values *T* were set with an increment of 0.1 °C.
- In the circuits *R_S_*/*V*_13_*S*_ and *R_S_*/*V*_13_*Stj*_, a resistor *R* with a nominal value equal to *R* = 7060 Ω and TCR*_R_* = 200 ppm/°C was used.
- The nominal supply voltage of the basic part of the measuring circuit was *V′* = 5 V.
- The ambient temperature (*T_a_*) of the standard resistors (*T_a_*_(*RStj*)_) and the rest of the ITT circuit under nominal conditions was 25 °C.
- Condition (4) was satisfied.

To show the effectiveness of the auto-calibration procedure, simulation tests were performed in the Mathcad program, enabling the selection of the degree of the polynomial (*J*) describing the ITT processing characteristics under nominal conditions with the smallest possible error Δ*_T_*. During the research, the temperature standards values (represented by *R_Stj_*) were also selected appropriately for each degree of the polynomial to achieve the lowest possible error values Δ*_T_*. The test results were consistent for two- and three-wire RTDs. For the nominal conditions, the following results were obtained:

- Δ*_T_* ∈ (−0.11, 0.097) °C for *J* = 2,
- Δ*_T_* ∈ (−0.0026, 0.0033) °C for *J* = 3,
- Δ*_T_* ∈ (−0.00013, 0.000093) °C for *J* = 4.

**Figure 2:**
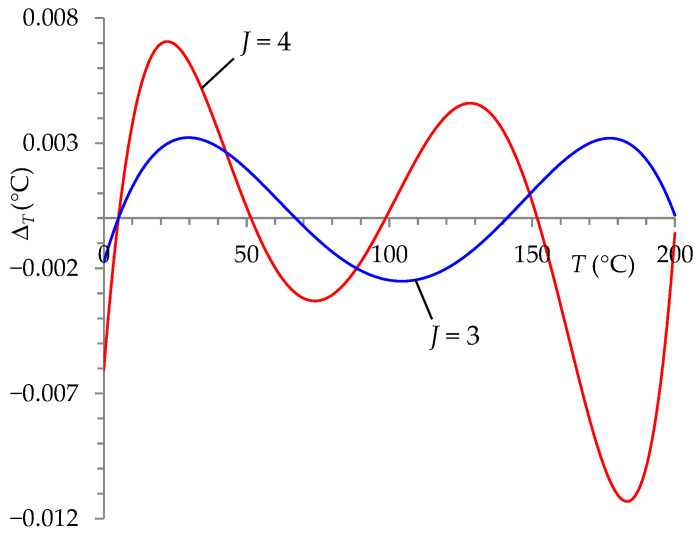
Temperature measurement error (Δ*_T_*) in nominal conditions for the ITT circuit with a voltage divider (*J* = 3) and the ITT with a Wheatstone bridge [13] (*J* = 4).

A polynomial of degree *J* = 3 was chosen for further research. For this polynomial, the error values of the presented ITT were smaller than those obtained for the transducer described in [13] (Figure 2) for *J* = 4 (Δ*_T_* ∈ (−0.011, 0.007) °C). Adopting such a solution (*J* = 3) allowed simplifying the auto-calibration circuit in the ITT, easier implementation of the auto-calibration procedure in μP, and shortening the running time of the procedure.

The implementation of the adopted degree of the polynomial (*J* = 3), for the assumed measurement range, was ensured by standard resistors (from *R_St_*_0_ to *R_St3_*) with the following nominal resistance values and corresponding standard temperatures (from *T_St_*_0_ to *T_St3_*):

- *R_St_*_0_ = 1020 Ω→*T_St_*_0_ = 5.121 °C;
- *R_St_*_1_ = 1260 Ω→*T_St_*_1_ = 67.192 °C;
- *R_St_*_2_ = 1540 Ω→*T_St_*_2_ = 141.110 °C;
- *R_St_*_3_ = 1760 Ω→*T_St_*_3_ = 200.392 °C.

It should be noted that the characteristics of the RTD and the *R_s_*/*V*_13_S_ circuit were strongly nonlinear. In the temperature range from 0 to 200 °C, such a detector had a linearity error of about 0.5%. The total linearity error of the *R_s_*/*V*_13_S_ circuit characteristic, taking into account the nonlinear RTD characteristic, was about 2%. The auto-calibration procedure minimized the influence of these nonlinearities on the *T* measurement result. For the assumed *J* = 3, the maximum relative error of the *T* measurement in nominal conditions was about 0.0017% (0.033 °C in the range from 0 to 200 °C). This result confirmed the effectiveness of minimizing the influence of the above-mentioned elements’ linearity errors.

**Figure 3:**
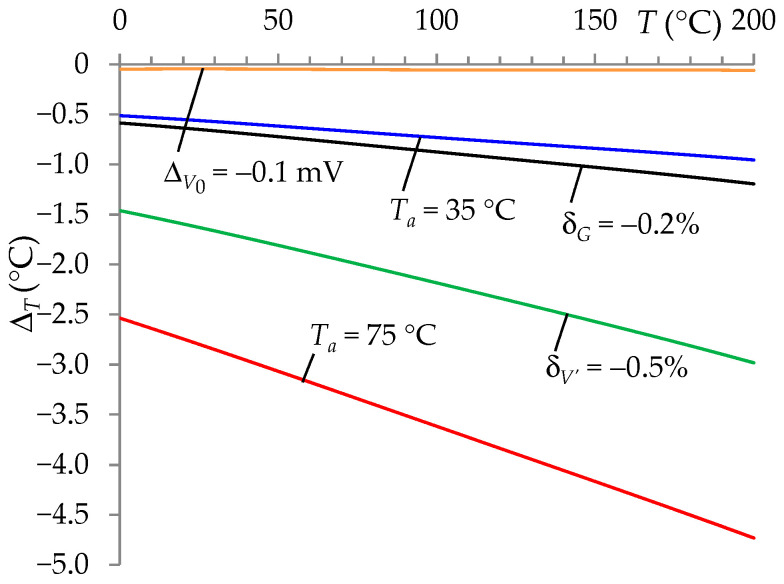
Temperature measurement error (Δ*_T_*) for the ITT without the auto-calibration procedure with the influence of ambient temperature (with values *T_a_* = 35 °C and *T_a_* = 75 °C) on the value of the resistor *R*, change in the supply voltage value (*V′*) by −0.5%, change offset voltage by −0.1 mV, and change in the A/D converter gain by −0.2%.

The effectiveness of the auto-calibration procedure for other sources of error or measurement uncertainty was tested in two cases. In the first case, *T* measurement errors were analyzed when this procedure was not used. In these tests, constant values of the coefficients in Equation (5) were assumed, which were determined for nominal conditions (the characteristic Δ*_T_* = *f*(*T*) was identical to that in Figure 2). However, much higher error Δ*_T_* values (Figure 3) were obtained in the following cases.

For example, see the following cases:

- If ambient temperature (*T_a_*) was changed from the nominal value of 25 °C to 35 °C affecting the resistor *R* with the value TCR*_R_* = 200 ppm/°C, then the maximum error of Δ*_T_* in the range 0–200 °C was −0.96 °C for *T_a_* = 35 °C and −4.8 °C for *T_a_* = 75 °C.
- If the *R_S_*/*V*_13_*S*_ and *R_Stj_*/*V*_13_*Stj*_ circuits’ supply voltage (*V′*) was changed by −0.5% concerning the nominal value, then the maximum error of Δ*_T_* was −3.0 °C.
- If the offset voltage (*V*_0_) was changed from 0 mV to −0.1 mV, then the maximum error of Δ*_T_* was −0.061 °C.
- If the gain error was changed from 0 to –0.2%, then the maximum error of Δ*_T_* was −1.2 °C.

Zero and gain errors came from the A/D converter.

In the second case, *T* measurement errors were analyzed using the auto-calibration procedure under the same conditions as in the first case. The proposed procedure effectively reduced the Δ*_T_* error value in the case of the influence of the ambient temperature *T_a_* on the resistor *R*. For *T_a_* = 75 °C, the Δ*_T_* error in the entire measurement range *T*, from 0 to 200 °C, varied within the range of −0.0025 °C to 0.0032 °C.

Similarly, after changing the following, the error Δ*_T_* over the entire measurement range for each of these cases varied within the same limits as in nominal conditions (from –0.0026 °C to 0.0033 °C):

- The voltage *V′* by –0.5%;
- The A/D converter offset voltage (*U*_0_) from 0 mV to –0.1 mV;
- The A/D converter gain error from 0 to –0.2%.

The results of the influence of changes in *T_a_*, *V′*, *V*_0_, and gain on the Δ*_T_* error, presented for all analyzed cases, proved that the auto-calibration procedure worked very well. Obtaining such a high efficiency of the auto-calibration procedure required ensuring that its execution time was short enough so that changes in environmental conditions had a negligible impact on the elements of the measurement track.

#### 2.2.2. The Influence of Measurement Channel Components on the Accuracy of Temperature Measurement

**Figure 4:**
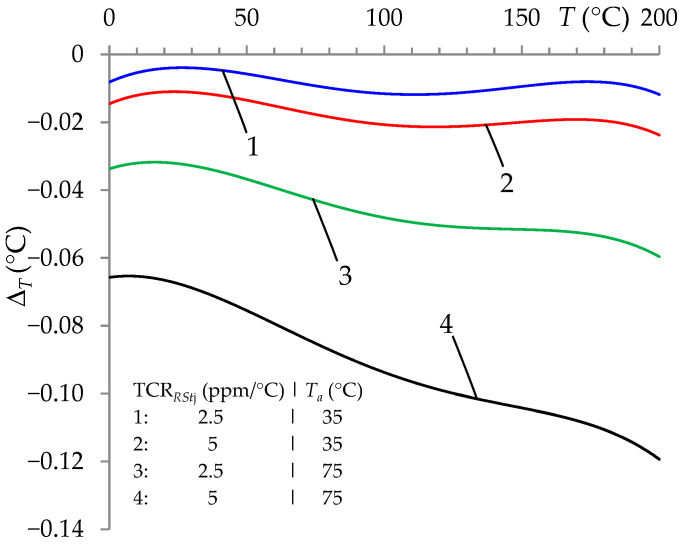
Temperature measurement error (Δ*_T_*) for the ITT with the auto-calibration procedure and with the influence of ambient temperature (with values *T_a_* = 35 °C and *T_a_* = 75 °C) on the values of *R_Stj_* resistors for TCR*_RStj_* = 2.5 ppm/°C and TCR*_RStj_* = 5 ppm/°C.

An important issue in the auto-calibration procedure is the influence of *T_a_* on changes in the values of the standard resistors *R_Stj_*, which significantly affects Δ*_T_* (Figure 4).

**Figure 5:**
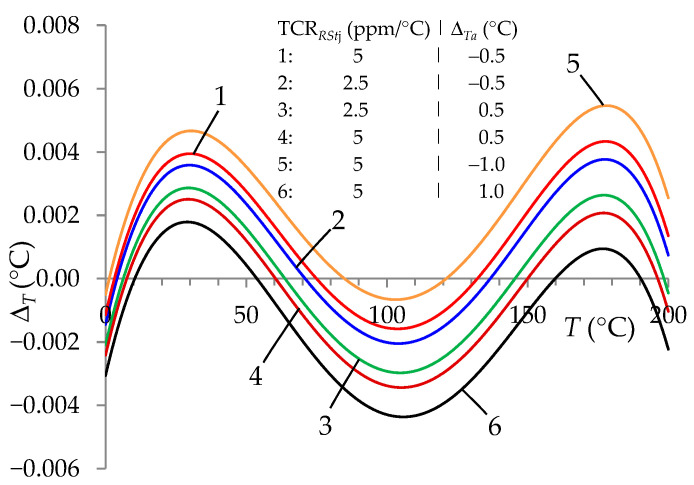
Temperature measurement error (Δ*_T_*) for the ITT with the auto-calibration procedure taking into account the measurement of the ambient temperature (*T_a_* = 75 °C) of standard resistors *R_Stj_* with an accuracy of ±0.5 °C and ±1 °C for TCR*_RStj_* coefficients equal to ±2.5 ppm/°C and ±5 ppm/°C.

Similarly to [13], it was proposed to measure the ambient temperature of the standard resistors (*T_a_*) and take it into account in the auto-calibration procedure. The effect of this action was a very significant reduction in the error value Δ*_T_* (Figure 5).

The choice of lower TCR*_RStj_* values and higher accuracy of measurement of the ambient temperature of the standard resistors (*T_a_*) only slightly changed the accuracy of *T* measurement. These were changes at the level of ten thousandths of °C.

It is assumed that temperature measurements are made under established environmental conditions. If these conditions change, then the measurement procedure should be carried out as briefly as possible so that changes in environmental conditions have a negligible impact on the components of the measurement channel.

**Figure 6:**
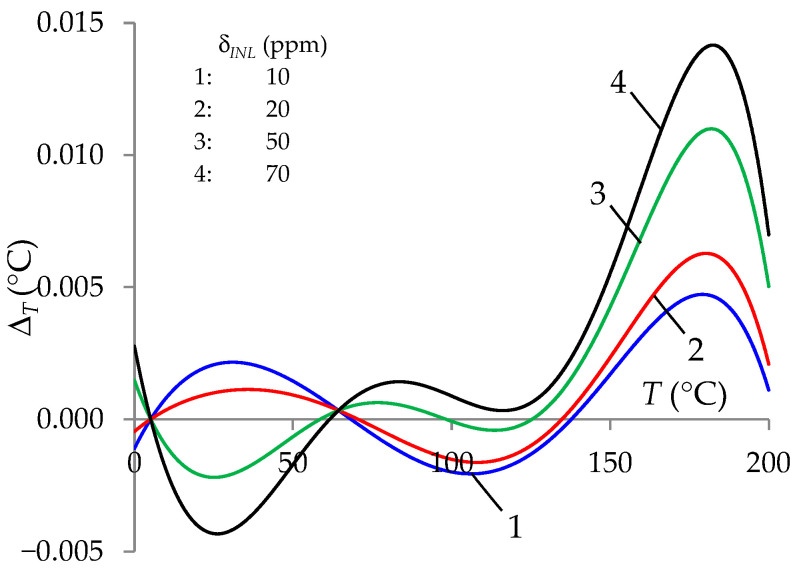
Temperature measurement error (Δ*_T_*) for the ITT with the auto-calibration procedure and with the influence of δ*_INL_* (10 ppm, 20 ppm, 50 ppm, and 70 ppm).

The accuracy of the *T* measurement using the ITT was also determined by the integral non-linearity (INL) of the A/D converter, which operated in the positive voltage range. For precise A/D converters, the INL characteristic in the entire range of positive voltage conversion could be similar to the half-period (e.g., MCP3550/1/3 converter [40]) or a periodic sine function (e.g., AD7766 converter [41]). The analyses of the test results taking into account the linearity error of the A/D converter in the ITT showed that higher error values were obtained for the periodic function. For this case, the values of the *V*_13′_ voltages that were the basis for calculating the measured value *T*, taking into account the INL of the A/D converter, were determined based on the developed relationship:

(8) $$V_{13}\prime =V_{13}\left(1+\delta_{INL}\sin\left(2\pi \frac{V_{13}}{V_{AD\max}}\right)\right),$$

where *V*_13_ takes the value of *V*_13_*S*_ or *V*_13_*Stj*_, respectively; δ*_INL_* is the limiting linearity error; and *V_AD_*_max_ is the range of the A/D converter. Figure 6 shows the influence of δ*_INL_* on the temperature *T* measurement error. The results of this test indicated the possibility of achieving the *T* measurement error below 0.01 °C with a linearity error δ*_INL_* less than 50 ppm.

Further, ITT research concerned the analysis of the uncertainty of temperature measurement, which was determined using the Monte Carlo (MC) method [42,43]. This method allowed for a more accurate estimate of the inaccuracy of the temperature measurement for the next influential quantities. For this method, quantities related to the scatter of the resistance values of the standard resistors (δ*_RStj_*), the scatter of the resistance values of the lines connecting *R_l_*__*S*_ and *R_l_*__*Stj*_ (Δ*_l_*__*Stj*,*S*_), the resolution of the A/D converter (Δ_A/D_), and noise (Δ_noise_), which may occur in the ITT circuit, were randomly generated *M* = 10^6^ times. For these data, uncertainties of temperature measurements (*U_T_*) were determined for a confidence level of 99%, based on the equation

(9) $$\Delta_{T}\left(i\right)=T_{MC}\left(i\right)-T,\;i=1,2,\ldots ,M,$$

where *T_MC_*(*i*) is the temperature determined on the basis of random data, according to the procedure described in Section 2.1.

**Figure 7:**
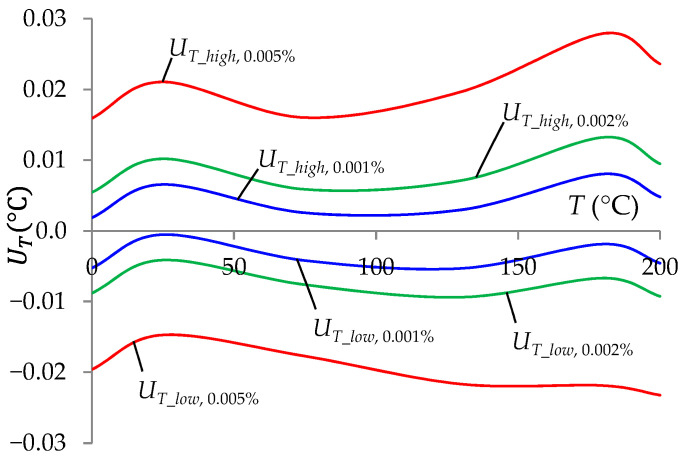
Temperature measurement uncertainty intervals at a 99% confidence level as a function of *T* for selected accuracies of standard resistors δ*_RStj_* (0.001%, 0.002%, and 0.005%).

Figure 7 presents the test results in the form of temperature measurement uncertainty intervals (*U_T_low_*, *U_T_high_*) as a function of the temperature measured for selected accuracies of the standard resistors. The obtained results indicated that achieving high accuracy in temperature measurement required the use of resistors with values known with high accuracy. For example, achieving *T* measurement uncertainty of approximately ±0.01 °C required the use of standard resistors with values known with an inaccuracy not greater than ±0.002%.

**Figure 8:**
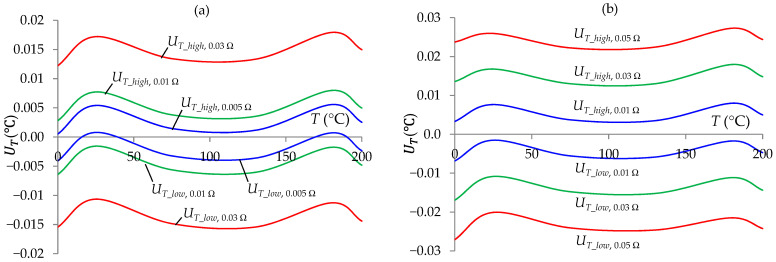
Temperature measurement uncertainty intervals at a 99% confidence level as a function of the measured temperature for selected values of errors: Δ_2*Rlw*_ (0.005 Ω, 0.01 Ω, and 0.03 Ω) (**a**) and Δ*_l_*__*Stj*,*S*_ (0.01 Ω, 0.03 Ω, and 0.05 Ω) (**b**).

As mentioned earlier, unfulfilled condition (4) caused inaccuracies in the temperature measurement. Such a situation occurred when the resistance of two wires of the sensor leading resistance *R_S_* to points 2–3 of the *R_S_*/*V*_13_*S*_ circuit (2*R_lw_*_1_3_) differed from the resistance of the compensation wire in points 3–4 of the *R_S_*/*V*_13_*Stj*_ circuit (2*R_lw_*_3_4_). The influence of the error of both resistances Δ_2*Rlw*_ on the result of the temperature measurement uncertainty is shown in Figure 8a. Moreover, the fulfillment of condition (4) is also influenced by the scatter of lines connecting the reference resistors and unequal resistances of the keys in the on state. The cumulative influence of the scatter Δ*_l_*__*Stj*,*S*_ of the resistance of the lines connecting the measurement channel components is shown in Figure 8b. The results of these tests indicated the possibility of obtaining *T* measurement uncertainty below the value of ±0.01 °C if Δ_2*Rlw*_ and Δ*_l_*__*Stj*,*S*_ was not greater than ±0.01 Ω.

**Figure 9:**
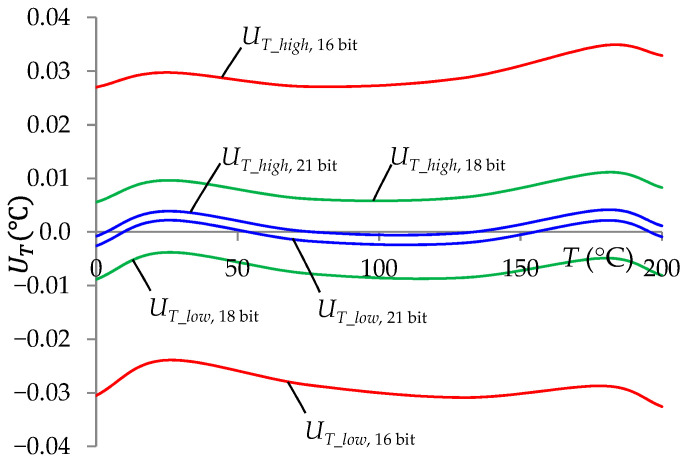
Temperature measurement uncertainty intervals at a 99% confidence level as a function of the measured temperature, for the adopted numbers of A/D converter bits.

Figure 9 shows the results of simulation tests determining the impact of the A/D converter resolution on the temperature *T* measurement uncertainty. The results of these tests indicated the possibility of achieving the *T* measurement expanded uncertainty at the level of ±0.01 °C using an A/D converter with a resolution of 18 bits.

**Figure 10:**
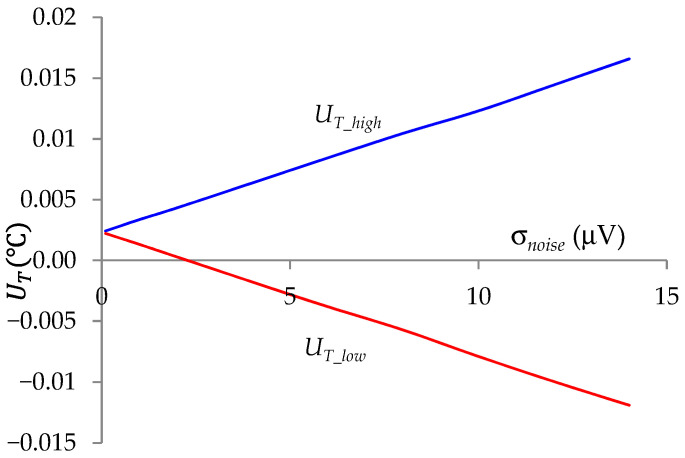
Uncertainty interval of temperature measurement *T* = 180 °C at a 99% confidence level as a function of the standard deviation of noise σ*_noise_*.

The study of the influence of Gaussian noise with standard deviation σ*_noise_* on the uncertainty of a single measurement *T* is illustrated in Figure 10. These were the results for the measurement case *T* = 180 °C, for which the highest uncertainties were obtained. This figure shows that it was possible to achieve *T* measurement uncertainty below ±0.01% with a standard deviation of noise less than 10 µV. It should be emphasized that for noise, the measurement uncertainty (resulting from the scatter of results) could be reduced by averaging a larger number of *T* measurements and/or using RC filters before the A/D converter [15].

In Figure 7, Figure 8, Figure 9 and Figure 10 the uncertainty intervals depending on the measured temperature are asymmetric. This is due to the influence of the systematic error for the nominal conditions (Figure 2) on the uncertainty range of the temperature measurement. This asymmetry is more visible with higher accuracy of temperature measurements, which is due to the greater influence of systematic error.

## 3. Realization of the Concept of an Intelligent Temperature Transducer

### 3.1. Materials 

A computer system that implements the ITT concept for measuring temperature in the range of 0–200 °C has been developed. The system was built to verify the correct operation of the ITT and to evaluate the uncertainty of temperature measurement for its exemplary implementation, taking into account the properties (test results) presented in Section 2. This measurement system consisted of the following components:

- Four 50 cm long copper wires: Two of them were used to lead *R_S_* to the measurement system (ITT), and the other two connected in series constituted a compensation loop (2*R_lw_*).
- A printed circuit board containing the *R_S_*/*V*_13_*S*_ circuit and auto-calibration circuit (*R_S_*/*V*_13_*Stj*_), as follows:
  − The *R_S_*/*V*_13_*S*_ circuit and auto-calibration circuit (*R_S_*/*V*_13_*Stj*_) were made of a resistor *R* = 7060 Ω with a tolerance of 1% and standard resistors (from *R_St_*_0_ do *R_St_*_3_) type RWE 0207 with a coefficient TCR*_RStj_* = 5 ppm/°C. The accuracy of the resistor *R* did not influence the measurement results *T* in the ITT. However, the accuracy of the standard resistors was important. For this reason, the values of the standard resistors were measured in a four-wire configuration with an Agilent 3458A multimeter with a limit error of δ*_RStj_* = ±0.002% (for a two-year calibration cycle). The following results were obtained: 1020.070 Ω, 1259.797 Ω, 1539.392 Ω, and 1759.317 Ω.
  − The *R_S_*/*V*_13_*S*_ circuit and auto-calibration circuit (*R_S_*/*V*_13_*Stj*_) contained reed switches (DIP05–1A72–12A [44]), which, unlike semiconductor switches, were characterized by low on-state resistance and relatively small scatter of their resistances.
  − The resistances *R_l_*__*S*_ and *R_l_*__*Stj*_ (including the resistances of the reed switches, sensor wire, compensation loop, and other component connections of the *R_S_*/*V*_13_*S*_ circuit and auto-calibration circuit), measured with a Thomson bridge, were each 0.12 Ω, and the scatter between these values (Δ*_l_*__*Stj*,*S*_) did not exceed 0.01 Ω.
- An Inmel SQ10 calibrator powering the *R_S_*/*V*_13_*S*_ circuit and the auto-calibration circuit with a voltage of *V′* = 5 V with a measured short-time instability of δ*_V′_* = ±10 ppm.
- An Agilent 3458A multimeter for measuring the *V*_13_ voltage with a measurement base error of Δ*_V_*_13_ = δ_1_*V*_13_ + Δ_1_ = 14∙10^−6^ *V*_13_ + 0.3 µV on a measuring range of ±1 V. It performed the tasks of an A/D converter (Figure 1). The digital voltmeter was made of many elements. In the measurement path, it contained a filter, an amplifier and an A/D converter. The complex construction of the voltmeter meant that its base error did not only result from the resolution of the A/D converter used. In the *T* measurement, the influence of the first component of the base error (δ_1_), which was of a constant nature, was minimized as a result of the auto-calibration procedure. The second component (Δ_1_), which was of a random nature, was more important. If this value were to be compared with the quantization error of the A/D converter used alone, it would correspond to a converter resolution of between 22 and 23 bits for the 0–1 V range. Additional errors of the voltmeter given by the manufacturer were as follows: the RMS noise was 0.02 ppm of range, the gain error was 0.01 ppm of reading, and nonlinearity was 0.3 ppm of reading and 0.1 ppm of range. These errors had a negligible effect on the accuracy of the *T* measurement at the level of 0.01 °C (see Section 2.2).
- A personal computer (PC) that performed the auto-calibration procedure, including the ability to calculate the temperature based on the results of voltage measurements obtained with the 3458A multimeter.

The ambient temperature of the standard resistors and the remaining part of the circuit (*T_a_*) during the measurements was 23 °C and did not exceed the error Δ*_Ta_* = ±1 °C.

### 3.2. Results of Simulation and Experimental Studies

The uncertainty of temperature measurement for the proposed system was estimated using the MC method. Similarly to the previous research (Section 2.2.2), the research was carried out in the Mathcad program for the assumed number of experiments *M* = 10^6^. Each *T_MC_*(*i*) temperature was determined using the auto-calibration procedure, based on the input values affecting the measurement results, i.e., δ*_RStj_*(*i*), TCR*_RStj_*(*i*), δ*_V′_*(*i*), δ_1_(*i*), Δ_1_(*i*), Δ*_Ta_*(*i*), and Δ*_l_*__*Stj*,*S*_(*i*). The values of these quantities were generated randomly (for *i* = 1…*M*) for the adopted rectangular probability distribution and the limit errors defined in the description of the computer circuit.

The voltages *V*_13_*S*_(*i*) (for the temperature sensor) and *V*_13_*Stj*_(*i*) (for the standard resistors) at the output of the *R_S_*/*V*_13_*S*_ and *R_Stj_*/*V*_13_*Stj*_ circuits were determined by the following formulas:

(10) $$V_{13\_S}\left(i\right)=k_{S}\left(i\right)V\prime^{*}\left(i\right)\left(1+\delta_{1}\left(i\right)\right)+\Delta_{1}^{*}\left(i\right),$$

(11) $$V_{13\_Stj}\left(i\right)=k_{Stj}\left(i\right)V\prime^{*}\left(i\right)\left(1+\delta_{1}\left(i\right)\right)+\Delta_{1}^{*}\left(i\right),$$

where

(12) $$k_{S}\left(i\right)=\frac{R_{S}+R_{l\_S}\left(i\right)}{R_{S}+R_{l\_S}\left(i\right)+R\left(i\right)},$$

(13) $$k_{Stj}\left(i\right)=\frac{R_{Stj}+R_{l\_Stj}\left(i\right)}{R_{Stj}+R_{l\_Stj}\left(i\right)+R\left(i\right)},$$

(14) $$R_{Stj}\left(i\right)=R_{Stj}\left(1+\delta_{R\;Stj}\left(i\right)\right)\left(1+TCR_{R\;Stj}\left(i\right)\Delta_{Ta}\left(i\right)\right),$$

(15) $$R_{l\_Stj}\left(i\right)=R_{l\_Stj}+\Delta_{l\_Stj}\left(i\right),$$

(16) $$R_{l\_S}\left(i\right)=R_{l\_S}+\Delta_{l\_S}\left(i\right),$$

(17) $$V\prime^{*}\left(i\right)=V\prime \left(1+\delta_{V\prime }^{*}\left(i\right)\right).$$

In addition, *R_S_* is the resistance of the temperature sensor calculated based on Equation (7). Moreover, * means that the values of a given input quantity were generated independently for each connected standard resistor and Pt1000 sensor in the auto-calibration and *R_S_*/*V*_13_*S*_ circuits.

**Figure 11:**
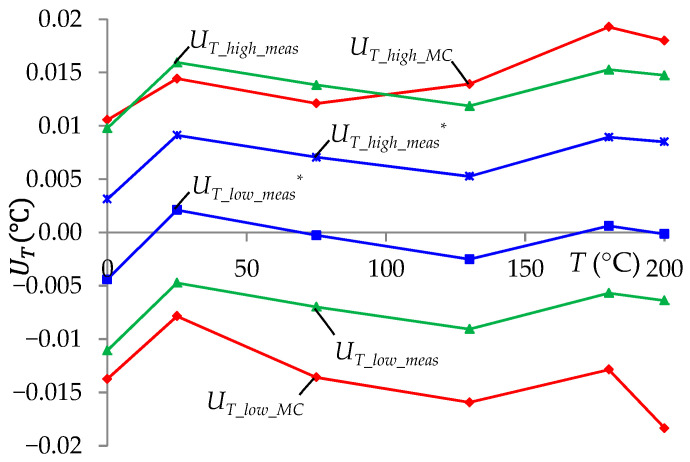
Measurement uncertainty interval at a 99% confidence level, as a function of the measured temperature: (*U_T_*__*low*_*MC*_, *U_T_*__*high*_*MC*_)—interval determined by simulation; (*U_T_*__*low*_*meas*_, *U_T_*__*high_meas*_)—interval determined experimentally; (*U_T_*__low_meas_^*^, *U_T_*__*high*_meas_^*^)—interval determined experimentally without taking into account Δ*_T_*__*set*_.

Based on the *T_MC_*(*i*) value and Formula (9), the *T* measurement uncertainty interval (*U_T_*__*low*_*MC*_, *U_T_*__*high*_*MC*_) for a confidence level of 99% was determined following the recommendations resulting from [42]. The obtained uncertainty results as a function of the measured temperature *T* are presented in Figure 11 with experimental results to compare. The limit values (endpoints) determined based on these tests were *U_T_*__*low*_*MC*_*min*_ = −0.019 °C and *U_T_*__*high*_*MC*_*max*_ = 0.02 °C. In addition, the temperature differences Δ*_T_* obtained in the simulation are presented in Table 1. The obtained results of the absolute values of Δ*_T_* were less than 0.004 °C. More detailed analyses of simulation test results showed that the influence of the voltmeter on the obtained uncertainty result *U_T_*__*high*_*MC*_*max*_ = 0.02 °C was negligibly small. The component of the base error of the voltmeter Δ_1_ = 0.3 µV, which was not minimalized as a result of the auto-calibration procedure, had a negligibly small influence in relation to the influences δ*_RStj_* = ±0.002% and Δ*_l_*__*Stj*,*S*_ = 0.01 Ω. Only an increase in the value of Δ_1_ to 7 µV (7 ppm in relation to the 1 V range) caused an increase in the uncertainty of the *T* measurement to *U_T_*__*high*_*MC*_*max*_ = 0.021 °C. It can therefore be assumed that for the ITT presented in Figure 1, the total influence of linearity, quantization, and noise errors of the A/D converter should not exceed 6 ppm if we want to keep the expanded measurement uncertainty at the level of 0.02 °C. These errors varied over the entire A/D converter range, so they were additive in nature (they added to the voltage *V*_13_), similarly as in the case of Δ_1_ of the voltmeter.

Experimental studies were conducted to verify the results of the simulation studies of the measurement system presented in Section 3.1. In the experimental tests, 10 temperature measurements were performed for each set standard temperature *T_set_* using the developed measurement system. Based on these measurements, the average *T_meas_* value and the standard uncertainty *u_T_*__*meas*_ were determined using the formulas

(18) $$T_{meas}=\frac{1}{N}\sum_{i=1}^{N}{T^{\prime }}_{i},$$

(19) $$u_{T\_meas}=\sqrt{\frac{1}{N\left(N-1\right)}\sum_{i=1}^{N}{\left({T^{\prime }}_{i}-T_{meas}\right)}^{2}},$$

where *N* = 10 is the number of measurements and *T_i_*’ is the *i*th temperature measurement result.

Then the error of temperature measurement was determined,

(20) $$\Delta_{T\_meas}=T_{meas}-T_{set}$$

and the expanded uncertainty interval at a 99% confidence level in which the true value of the measured temperature was located. The limits of this interval (endpoints) were determined based on the Guide [45], from the formulas

(21) $$U_{T\_high\_meas}=\Delta_{T\_meas}+\sqrt{3}\cdot 0.99\sqrt{u_{T\_meas}^{2}+{\left(\frac{\Delta_{T\_set}}{\sqrt{3}}\right)}^{2}},$$

(22) $$U_{T\_low\_meas}=\Delta_{T\_meas}-\sqrt{3}\cdot 0.99\sqrt{u_{T\_meas}^{2}+{\left(\frac{\Delta_{T\_set}}{\sqrt{3}}\right)}^{2}},$$

where Δ*_T_set_* is the error of the standard temperature setting, and $\sqrt{3}$·0.99 is the coverage factor for the uncertainty Δ*_T_set_*/$\sqrt{3}$ with a rectangular distribution, which dominates in the measurement.

When performing measurements, the standard temperatures *T_set_* were set by connecting standard resistors instead of the temperature sensor. The standard resistors represented the *R_S_* resistances, thereby eliminating the influence of RTD inaccuracies on ITT studies. Before being connected to the measurement circuit, the resistance of each standard resistor was measured with four wires using an Agilent 3458A multimeter to obtain the highest possible accuracy of the *T_set_* setting calculated from Formula (7). Based on the measurement error of the resistance of the resistors representing the resistance of the *R_S_* sensor, which was ±0.002%, the temperature error Δ*_T_*__*set*_ was estimated at ±0.01 °C.

The results of the calculated measurement uncertainties are presented in Figure 11. The experimentally obtained limit values in the entire range of measured temperatures did not exceed the values *U_T_*__*low*_*meas*_*min*_ = −0.012 °C and *U_T_*__*high*_*meas*_*max*_ = 0.016 °C. These were the values that did not exceed the limit values obtained by simulation. This indicated a high agreement between the simulation and the experimental results. It is worth noting that the error Δ*_T_set_* = 0.01 °C had a large impact on the measurement uncertainty results (*U_T_*__*low*_*meas*_, *U_T_*__*high*_*meas*_). If this component was not considered in the measurement uncertainty calculations ((21), (22)), then the obtained uncertainty values (*U_T_*__*low*_meas_^*^, *U_T_*__*high*_meas_^*^) were much smaller than the uncertainties determined by simulation (Figure 11) in the entire range of the measured temperature.

This may mean that the real measurement inaccuracy is smaller than that resulting from the limit errors of the measurement uncertainty sources. This is also an indication to the authors that in further research aimed at increasing the accuracy of temperature measurement, it will be necessary to use temperature standards with even greater accuracy than 0.01 °C. The effectiveness of the applied auto-calibration procedure was confirmed by the summary of the results of temperature differences Δ*_T_* and Δ*_T_*__*meas*_ obtained as a result of simulation and experimental tests in Table 1. The differences in the results were at the level of thousandths of °C.

## 4. Conclusions

The presented results of the simulation and experimental studies showed that the auto-calibration procedure presented in [38] could also be used in a temperature transducer in which the *R_S_*/*V* circuit was in the form of a resistive divider. The advantage of the new solution was primarily the higher accuracy of temperature measurement compared with a transducer with a Wheatstone bridge [13] (Appendix A). Other advantages of the new transducer were the simpler structure of the *R_S_*/*V* circuit and the possibility of using a lower degree polynomial *J* = 3 in the auto-calibration procedure compared with the transducer with a bridge in which *J* = 4 was adopted. Consequently, this translated into the possibility of reducing the number of standard resistors in the ITT and easier software implementation of the procedure due to the smaller number of required coefficients in Equation (3).

The obtained simulation test results showed that the new ITT could achieve a *T* measurement accuracy of 0.0033 °C under nominal conditions. It was resistant to the influence of RTD and *R_S_*/*V*_13_ circuit nonlinearity, zero and gain errors, and supply voltage changes. The *T* measurement accuracy was influenced by the parameters of standard resistors, resistance scatter of connecting lines (*R_l_*__*S*_, *R_l_*__*Stj*_), resolution and nonlinearity of the A/D converter, and noise in the ITT.

The obtained results of the simulation and experimental tests of the developed measurement system, performing ITT tasks, showed that the new ITT enabled the processing of the Pt1000 sensor resistance with an accuracy of 0.02 °C. This could be achieved by using the following in the ITT:

- Four standard resistors (*R_Stj_*) with an accuracy of ±0.002% and TCR*_RStj_* = 5 ppm/°C along with a sensor measuring the ambient temperature with an error of no more than ±1 °C;
- A connection circuit in which the line resistances fulfill the condition *R_l_*__*S*_ = *R_l_*__*Stj*_ with an error of no more than ±0.01 Ω;
- A circuit for converting analog voltage *V*_13_ to a digital form with a total error (resulting from noise, quantization, and nonlinearity) no greater than 6 ppm.

It should be mentioned that the use of standard resistors with an accuracy of ±0.002% in the ITT does not require the purchase of expensive resistors with a tolerance of ±0.002%. As presented in the work, these may be resistors with a much smaller tolerance, the resistances of which should be measured with an accuracy of ±0.002%. The 3458A multimeter used for this purpose makes it possible to obtain this accuracy during its two-year calibration cycle. More frequent calibration allows for even higher accuracies, e.g., ±0.00125% for 1 year.

The developed ITT makes it possible to perform accurate *T* measurements using two- and three-wire platinum RTDs. Achieving high temperature measurement accuracy is possible by minimizing the influence of lead wire resistance and nonlinearity of the RTD and also nonlinearity, offset, gain error, supply voltage changes, and ambient temperature of the ITT on the temperature measurement result. The obtained test results indicate that the ITT can cooperate with the RTD about accuracy corresponding to class AA [4].

The research results presented in the paper indicate that it is possible to achieve ITT accuracy below 0.02 °C, which is required, for example, in calibration laboratories. This means, above all, solving in the future the problem of being able to reproduce standard temperatures with higher accuracy. Moreover, further research is required to develop an *R_S_*/*V* circuit in which the spread of the resistance values of the *R_l_*__*S*_ and *R_l_*__*Stj*_ connection lines would be smaller than ±0.01 Ω.

## Figures and Tables

**Table 1:** Temperature differences Δ*t* and Δ*_T_meas_* obtained in the simulation and experimental tests for the ITT with the voltage divider.

*T_set_* (°C)	Δ*_T_* (°C) (Simulation)	Δ*_T_meas_* (°C) (Measurement)
0	0.0028	−0.00066
25	0.0032	0.0056
75	−0.00087	0.0035
130	−0.0012	0.0014
180	0.0032	0.0048
200	0.000057	0.0042

## Data Availability

The data used to support the findings of this study are available from the authors upon request.

## Appendix A

This section presents a comparison of the test results of the new transducer (with a resistive divider) with the results obtained for a transducer with a Wheatstone bridge [13]. The same auto-calibration procedure, presented in Section 2, was used in both circuit solutions. However, in the previous solution (with a bridge), a polynomial of degree *J* = 4 was used in the procedure, and in the new one, *J* = 3 was used. The obtained test results in the form of maximum absolute errors Δ*_T_*__*max*_ or uncertainty *U_T_*__*max*_ for both circuit solutions and various measurement cases, described in Section 2 and Section 3, are presented in Table A1.

Analyzing the test results, it can be noticed that in each of the tests performed, the new transducer had a higher *T* measurement accuracy than the transducer presented in [13].

**Table A1:** Results of Δ*_T_max_* and *U_T_*__*max*_ at a 99% confidence level obtained in various tests of the new transducer and presented in [13].

Type of Test	Parameters	New ITT	Previous ITT
		Δ*_T__*_max_ or *U_T_max_* (°C)	
Tests under nominal conditions	*T* = 0…200 °C	0.0033 *	0.011 *
Tests for changed ambient temperature	*T_a_* = 75 °C; TCR*_RStj_* = 5 ppm/°C; Δ*_Ta_*_(*RStj*)_ = 1 °C; *T* = 0…200 °C	0.0055 *	0.014 *
Analysis of the influence of tolerances on standard resistors	δ*_RStj_* = 0.002%; *T* = 0…200 °C	0.014 **	0.026 **
Analysis of the influence of the resistance of the connecting circuit	Δ*_l_*__*Stj,S*_ = 0.01 Ω; *T* = 0…200 °C	0.0080 **	0.017 **
Analysis of the influence of A/D converter resolution	*B* = 18 bit; *T* = 0…200 °C	0.012 **	0.027 **
Noise impact analysis	σ*_noise_* = 14 µV; *T* = 0…200 °C	0.017 **	0.067 **
Comprehensive simulation test	TCR*_RStj_* = 5 ppm/°C; Δ*_Ta_* = 1 °C; δ*_RStj_* = 0.002%; *T_a_* = 23 °C; Δ*_V_*_13_ = 14∙10^–6^ *V*_13_ + 0.3 µV; *T* = 0…200 °C; Δ*_T_*__*set*_ = 0.01 °C	0.020 **	0.029 **
Comprehensive experimental test		0.016 **	0.021 **
Comprehensive experimental tests without taking into account Δ*_T_set_*		0.0091 **	0.013 **

*—value of error Δ*_T__*_max_; ^**^—value of uncertainty *U_T_max._*
